# The Effectiveness of Nasal Conformers in Patients with Unilateral Cleft Lip and Palate Following Primary Cheiloplasty: A Systematic Review and Meta-Analysis

**DOI:** 10.1055/a-2572-6342

**Published:** 2025-07-23

**Authors:** Jutharat Chimruang, Saran Worasakwutiphong, Ratchawan Tansalarak

**Affiliations:** 1 Department of Preventive Dentistry, Faculty of Dentistry, Naresuan University, Phitsanulok, Thailand; 2 Naresuan University Cleft and Craniofacial Center, Phitsanulok, Thailand; 3 Department of Surgery, Faculty of Medicine, Naresuan University, Phitsanulok, Thailand

**Keywords:** Cleft Lip, nasal stents, systematic review, meta-analysis

## Abstract

**Background:**

This study aims to evaluate the effectiveness of nasal conformers in patients with unilateral cleft lip and palate (UCLP) following primary cheiloplasty.

**Methods:**

We conducted a comprehensive search in PubMed, EMBASE, the Cochrane Central Register of Clinical Trials (CENTRAL), and EBSCO Open Dissertation from inception to November 2023. We included three retrospective and one prospective cohort studies evaluating the effect of postoperative nasal conformers after primary cheiloplasty. Data extraction and risk of bias assessment were performed using the Risk of Bias in Non-Randomized Studies of Intervention (ROBINS-I) tool. A meta-analysis was conducted using a random-effects model to pool data and determine overall effect sizes with a 95% confidence interval (CI). The mean difference (MD) was calculated for nostril height and nostril width outcomes. The study was registered with PROSPERO (CRD42024511395).

**Results:**

The four included studies represented 83 treated patients and 84 controls. The overall quality of studies was moderate, as assessed by the ROBINS-I tool. Meta-analysis results showed that nostril width was wider on the cleft side compared with controls (MD = 1.10; 95% CI: 0.64, 1.56), while nostril height was lower on the cleft side (MD = −0.73; 95% CI: −1.20, −0.26). Ratios for nostril width and nostril height were generally closer to symmetry.

**Conclusion:**

This study suggests that nasal conformers may improve nasal symmetry in patients with UCLP following primary cheiloplasty. Further research, including randomized controlled trials and long-term follow-up studies, is needed to confirm these findings and refine the use of nasal conformers in clinical practice.

## Introduction

Correcting unilateral cleft lip nasal deformities is crucial for aesthetic reasons, with primary concerns focusing on alar cartilage luxation. Determining the optimal management approach for these deformities remains uncertain due to the challenges and limited surgical options. Restoring lip and nose symmetry is essential for improving patients' self-esteem and overall quality of life, leading to the adoption of a variety of techniques, including nasoalveolar molding (NAM), lip adhesive tapes, and nasal stents/conformers before and after surgery. 1 2 3 4 Preoperative nasal conformers and NAM can improve nasal symmetry before primary cheiloplasty in patients with unilateral cleft lip and palate (UCLP). 5 6 Despite these interventions, maintaining the corrected nose shape postcheiloplasty is challenging due to scar contraction, emphasizing the ongoing need for postoperative care using tools like nasal retainers. 7 8 9

**Fig. 1:**
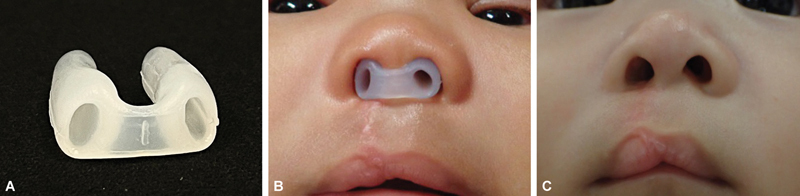
Illustration of nasal conformer and its postoperative application and outcomes. ( **A** ) Example of a nasal conformer. ( **B** ) Application of a nostril retainer postsurgery, maintaining nasal contour, preventing nostril collapse, and ensuring airway patency. ( **C** ) Posttreatment results showing symmetrical and well-shaped nostrils.

The clinical impact of postoperative nasal conformers following primary cheiloplasty in patients with UCLP remains a subject of debate in craniofacial surgery. Nasal conformers are devices, either prefabricated or custom-made, designed to help maintain the shape and symmetry of the nasal passage after primary cheiloplasty. These devices are typically inserted postoperatively to support nostril molding, reduce nasal asymmetry, and prevent collapse during the healing process. Figure 1 demonstrates an example of a nasal conformer used in patients following primary cheiloplasty. Written informed consent was obtained from the patient or their guardian for the use of their photograph and clinical information in this study.

While some studies suggest these devices improve nasal shape, aesthetics, and long-term symmetry, 9 10 11 the outcomes across studies have varied, partly due to differences in methodologies and patient demographics. Patients using postsurgical nostril retainers have shown significantly better nasal shape and symmetry, with improvements in alar width, columella length, and nostril aperture dimensions. 12 13 Nevertheless, maintaining nasal symmetry long-term remains a challenge, with relapse often occurring within the first year postoperatively. 10 14 Despite these challenges, nasal conformers contribute to improved nasal shape and aesthetic outcomes, potentially enhancing the quality of life for UCLP patients. 12 Further standardized research is needed to establish definitive conclusions on the effectiveness of these interventions.

Although scientific evidence regarding its efficacy remains limited, postoperative nasal conformers are used to maintain surgical outcomes and potentially prevent nasal deformity recurrence. However, due to the lack of substantial high-quality evidence, this study systematically reviews the impact of nasal conformers after primary cheiloplasty in patients with UCLP, aiming to provide clear insights into their effectiveness and contribution to improving nasal outcomes.

## Methods

### Literature Search Methodology

A systematic review was conducted according to the Cochrane Collaboration guidelines for a systematic review of interventions 15 and following the PRISMA 2020 statement. 16 Electronic searches of scientiﬁc literature were performed using PubMed, EMBASE, Cochrane Central Register of Clinical Trials (CENTRAL), and EBSCO Open Dissertation up to November 2023, with no language restrictions. The search terms included two domains: (1) “Cleft lip,” which encompassed the following terms: “Cleft lip” OR “Cleft lip”[MeSH] OR “Hare lip” OR “Mouth cleft” OR “Oral cleft” OR “Orofacial cleft”; and (2) “Stent,” which included the terms: “Stent” OR “Stents”[MeSH] OR “Conformer” OR “Retainer” OR “Creator.” A detailed search strategy for each database is provided in Supplementary Table S1 (available in the online version only). To ensure comprehensive coverage, additional search techniques such as citation tracking and snowballing were also employed using Scopus. The study protocol was registered on PROSPERO (ID: CRD42024511395).

### Selection Criteria

The PICO (Patient, Intervention, Comparison, Outcome) framework was used to construct this systematic review with no language restrictions. The PICO is outlined as follows:

Patient: Patients with UCLP who underwent primary cheiloplasty.

Intervention: The use of postoperative nasal conformers on the cleft side following primary cheiloplasty.

Comparison: Patients who used postoperative nasal conformers on the non-cleft side or who did not use postoperative nasal conformers following primary cheiloplasty.

Outcome: Nasal anthropometric parameters as measured by at least two of the following clinical outcomes: nostril width, nostril height, nostril width ratio, nostril height ratio, columella length, and alar base width.

All human studies were included in the review. Due to the scarcity of randomized controlled trials (RCTs) on this subject, non-randomized controlled studies were also considered. The initial screening of article selections was performed by two independent reviewers (R.T. and J.C.) according to inclusion and exclusion criteria. After removing duplicates, the reviewers independently screened article titles and abstracts to identify eligible studies. Subsequently, the full texts of potential studies were reviewed to confirm eligibility. Disagreements regarding study inclusion or exclusion were resolved through consensus or consultation with the third reviewer (S.W.).

### Data Extraction

The following information was independently extracted using standardized form by the same reviewers: (1) authors, (2) year of publication, (3) country, (4) study design, (5) sample size, (6) age at follow-up, (7) presurgical device, (8) surgical technique, (9) type of nostril retainer, (10) duration of using nostril retainer, (11) measurement method, (12) results, (13) source of funding, and (14) conflict of interest.

### Assessment of Methodological Quality

Two independent reviewers (R.T. and J.C.) assessed the methodological quality of the included studies using the Risk of Bias in Non-Randomized Studies of Intervention (ROBINS-I) tool. 17 This assessment encompassed seven domains, including (1) bias due to confounding, (2) bias in selection of participants into the study, (3) bias in classification of interventions, (4) bias due to deviations from intended interventions, (5) bias due to missing data, (6) bias in measurement of outcomes, and (7) bias in selection of the reported results. The overall risk of bias across these domains was classified as low, moderate, serious, critical, or no information. Any disagreements were resolved through consensus after discussion with the third reviewer (S.W.).

### Statistical Analysis and Meta-Analysis

Nasal anthropometry data from all studies were pooled using the Mantel–Haenszel random-effects meta-analysis to determine the overall effect size with a 95% confidence interval (CI). 15 The random-effects model was chosen to account for potential heterogeneity among the included studies. The mean difference (MD) was calculated for nostril height and nostril width effects. Heterogeneity was evaluated using the chi-square test and *I* ^2^ statistic, with an observed *I* ^2^ value of 30% to 60% considered moderate heterogeneity. 15 Interpretation of *I* ^2^ thresholds took into consideration the magnitude and direction of effects, as well as the strength of evidence of heterogeneity. All analyses were performed by using ReviewManager (RevMan) version 5.4.

## Results

### Study Selection

**Fig. 2:**
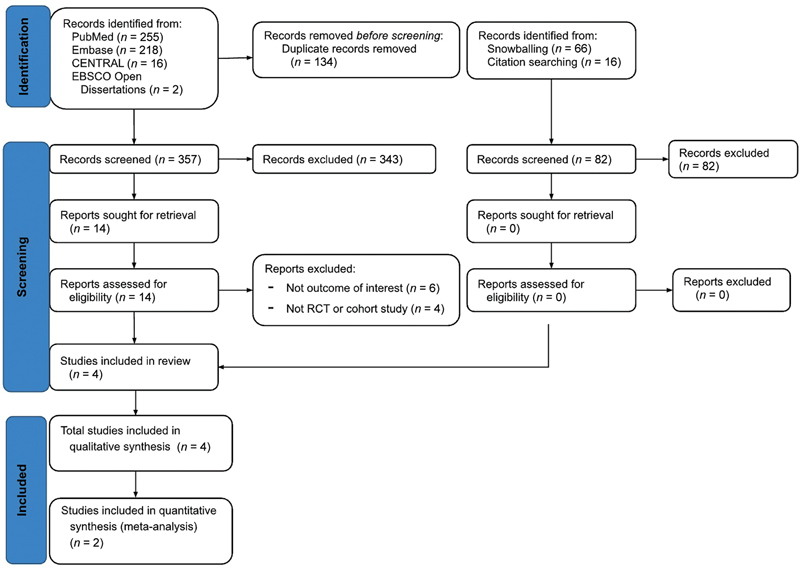
PRISMA flow diagram for the identification and selection of studies. CENTRAL, Cochrane Central Register of Clinical Trials; RCT, randomized controlled trial.

The PRISMA diagram illustrates the selection process. The electronic database search initially yielded 491 records, which were reduced to 357 articles after removing duplicates. After screening titles and abstracts, 343 articles were excluded, leaving 14 studies for full-text examination. An additional 10 studies were excluded based on the reasons described in Fig. 2 . Supplementary Table S2 (available in the online version only) lists the excluded studies after full-text review, categorized as follows: six studies had no outcome of interest, and four studies were not RCTs or cohort studies. Ultimately, four studies 18 19 20 21 were selected for qualitative synthesis, and two studies 20 21 were selected for quantitative synthesis.

### Characteristics of Included Studies

A summary of the included studies is provided in Tables 1 and 2 , detailing their characteristics, interventions, and outcomes. The review included three retrospective and one prospective cohort studies, comprising 83 treated patients and 84 controls. Nasal conformer appliances varied, including preformed silicone nasal conformers, nasal strapping tape, and silicone postnostril retainers, such as those manufactured by Koken Co., Ltd., Tokyo, Japan. While all studies evaluated postoperative nasal conformer use, the methodologies differed slightly, reflecting the complexity of cleft care across diverse clinical settings.

**Table 1:** The detailed characteristics of the included studies

Study	Study type	Number of patients	Age	Cleft type	Presurgical device	Surgical technique of cheilorhinoplasty	Nasal conformer type	Retention of nasal conformer	Follow up	Source of funding	Conflict of interest
Chang et al. 18 (2018)	Retrospective cohort study	36	Unreported	Unilateral complete cleft of lip and primary palate without cleft of the secondary palate	Yes and none	Rotation-advancement cheiloplasty and Tajima method	Silicone nasal stents	6 months	Unreported	None	None
Funayama et al. 19 (2019)	Retrospective cohort study	63	Unreported	Unilateral complete cleft lip and palate	None	Closed nasal cartilage dissection during lip repair	Silicone postnostril retainers (Koken Co., Ltd. Tokyo, Japan)	6 months	Unreported	None	None
Waewsanga et al. 20 (2021)	Prospective cohort study	16	5 months 15 days	Unilateral cleft lip and palate	Unreported	Unreported	Preform nasal creator	6 months along 24 hours	1, 3, and 6 months	National Research Council of Thailand	None
Al-Qatami et al. 21 (2022)	Retrospective cohort study	50	12 months, 8 days	Unilateral complete cleft lip and palate	Yes	Extended Mohler technique	A preformed, medical-grade, soft silicone nostril retainer (Stryker, Kalamazoo, MI, USA)	3 months 19 days	Every 2 weeks	None	None

**Table 2:** Outcomes of the included studies evaluating nasal symmetry with objective metrics

Study (control group: Y/N)	Number of patients (treatment/control)	Outcome measured	Direct versus indirect metric of measured	Baseline of time point measured	Final time point measured	Conclusion (statistical significance: Y/N)
Chang et al. 18 (Y)	36 (36/36)	Nostril width ratio and nostril height ratio	Indirect by two-dimensional facial photographs	Unreported	Age 3–9 years	The measurements indicated symmetry between the cleft and non-cleft sides, with all ratios being close to 1 ( *p* > 0.05). The nostril width was wider on the cleft side (NW/NW′ = 1.33), whereas the nostril height was shorter on the cleft side (NH/NH′ = 0.85) (N).
Funayama et al. 19 (Y)	63 (7/6)	Nostril width, nostril height, nostril width ratio, nostril height ratio, and columella length	Indirect by two-dimensional facial photographs	Unreported	Age 4 years	Nostril height was higher in the experimental group than in the control group and showed a statistically significant difference. The nostril height ratio was significantly greater in the experiment group, whereas the nostril width ratio did not differ significantly (Y, N).
Waewsanga et al. 20 (Y)	16 (16/16)	Nostril width, nostril height, and alar base width	Indirect by three-dimensional photographs	Postop 1 day(5 months, 15 days)	Postop 6 months	By T4, there were no significant differences in nostril height and width between the affected and non-affected sides (N).
Al-Qatami et al. 21 (Y)	50 (24/26)	Nostril width, nostril height, columella length, and alar base width	Direct by model casts	Unreported	Age 12 months 8 days	Nasal anthropometric measurements on the cleft side were statistically significantly better in group 1 (postsurgical nostril retainer) than in group 2 (control) across all measures ( *p* < 0.05). Additionally, within group 1, the cleft side measurements showed statistically significantly lower values for nostril height, higher values for nostril width, alar base width, and no significant difference in columella length (Y, N).

The nasal conformer treatment duration ranged from 3 months and 19 days to 6 months, with varied follow-up intervals for retention. Nasal symmetry was assessed using direct and indirect anthropometric measurements. Nostril width and nostril height were consistently evaluated, with nostril width measured as the widest horizontal distance between the nostril's inner medial and lateral borders, and nostril height measured from the lateral border at the base of the columella to the highest point of the nostril. However, two studies provided incomplete descriptions of their protocols, and one study did not use a presurgical device like NAM, which may influence baseline conditions. Additionally, variability in patient demographics, including age at surgery, follow-up duration, and unreported cleft severity, likely contributed to differences in outcomes among the studies.

### Assessment of Methodological Quality

The risk of bias was evaluated using the ROBINS-I tool, 17 with all studies classified as having moderate bias ( Table 3 ). Considering the ROBINS-I classification, we found overall risk at a moderate level and each category at low to moderate levels. The study objectives, main outcomes, patient characteristics and interventions were clearly described by all included studies. However, due to the nature of the intervention, blinding of the participants was not possible in most studies. All studies attempted to blind the outcome assessors.

**Table 3:** Risk of bias assessment by domains and overall risk levels using the ROBINS-I tool

Study	Preintervention		At intervention		Postintervention			Overall bias
	Bias due to confounding	Bias in selection of participants in the study	Bias in classification of intervention	Bias due to deviations from intended interventions	Bias due to missing data	Bias in measurement of outcomes	Bias in selection of the reported results	
Chang et al. 18	Moderate	Low	Low	Low	Low	Low	Low	Moderate
Funayama et al. 19	Moderate	Low	Low	Low	Low	Low	Low	Moderate
Waewsanga et al. 20	Moderate	Low	Low	Low	Low	Low	Low	Moderate
Al-Qatami et al. 21	Moderate	Low	Low	Low	Low	Low	Low	Moderate

### Meta-Analysis Findings

**Fig. 3:**
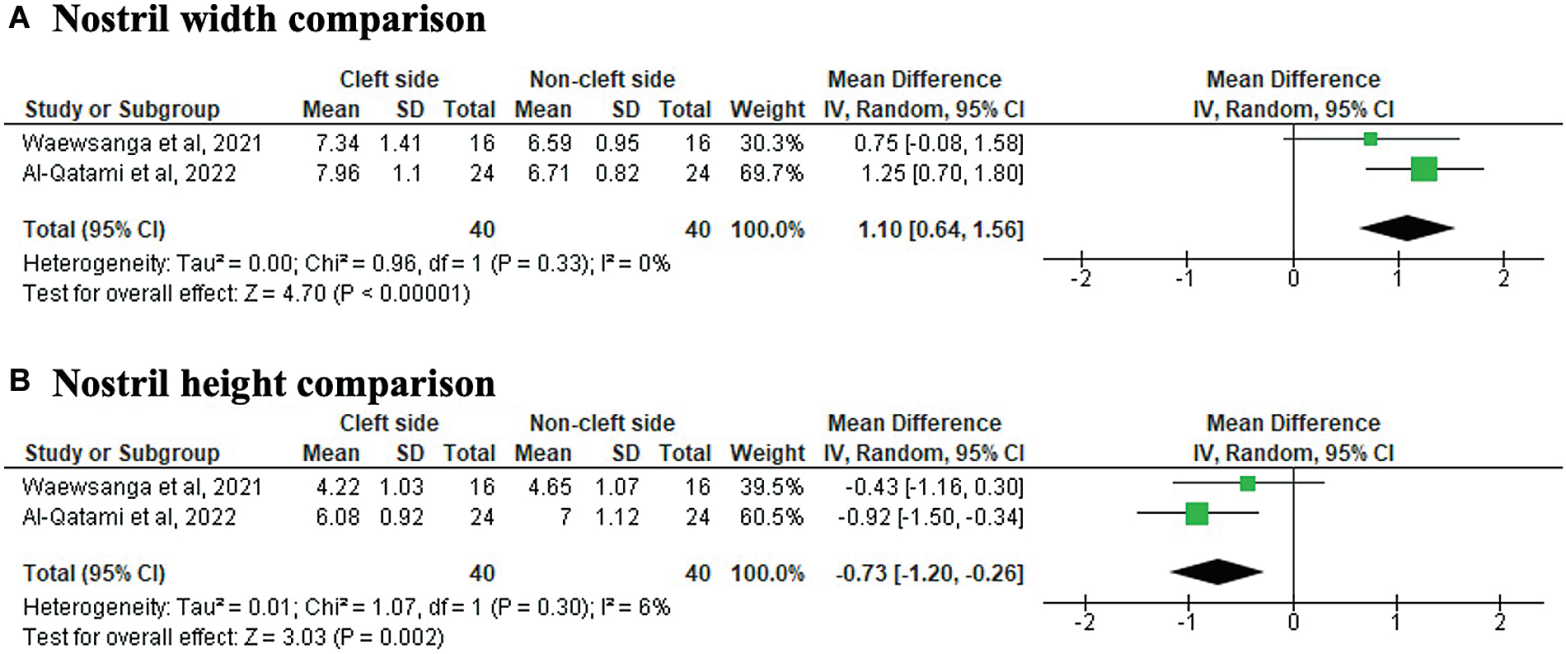
Forest plot comparing nasal anthropometry parameters between the cleft side and the non-cleft side postoperative nasal conformers; ( **A** ) nostril width and ( **B** ) nostril height. CI, confidence interval; SD, standard deviation.

We were able to identify the pooled estimate using a meta-analysis approach for only nostril width and nostril height outcomes from two studies. 20 21 For nostril width, the results associated that the cleft side group had a wider nostril width compared with the control group (non-cleft side; MD = 1.10; 95% CI: 0.64, 1.56), with no heterogeneity ( *I* ^2^ = 0%; Fig. 3A ). For nostril height, the cleft side showed a significant reduction compared with the control group (MD = −0.73; 95% CI: −1.20, −0.26), with minimal heterogeneity ( *I* ^2^ = 6%; Fig. 3B ). These findings on nostril width and nostril height suggest that residual asymmetry remains. The difference in measurements between the cleft and non-cleft sides was +1.10 mm of nostril width and −0.73 mm of nostril height.

The absence of heterogeneity ( *I* ^2^ = 0% for width) and minimal heterogeneity ( *I* ^2^ = 6% for height) suggest that the pooled estimates were consistent across studies. However, it is essential to interpret these findings with caution, as the limited number of studies ( *n* = 2) reduces the robustness of the statistical analysis and may not fully represent the broader population. The clinical significance of these findings, particularly the observed asymmetry, warrants further investigation in the context of functional and aesthetic outcomes.

### Descriptive Findings

#### Nostril Width

All four studies assessed the effect of nasal conformers on nostril width ( Table 4 ). Three studies consistently reported significant improvements in nostril width with nasal conformers, while one study reported a wider nostril width on the cleft side, contrary to the general trend. 18

**Table 4:** Summary of descriptive findings on nostril width, nostril height, and symmetry ratios

Study	Nostril width findings	Nostril height findings	Symmetry ratio findings
Chang et al. 18 (2018)	Wider on cleft side, contrary to general trend	Shorter on cleft side compared with non-cleft side	Ratios closer to 1, indicating better symmetry
Funayama et al. 19 (2019)	Significant improvement with nasal conformers	Significant improvement with nasal conformers	Significant difference in nostril height ratio but not width ratio
Waewsanga et al. 20 (2021)	Significant improvement with nasal conformers	Significant improvement with nasal conformers	Not explicitly reported
Al-Qatami et al. 21 (2022)	Significant improvement with nasal conformers	Significant improvement with nasal conformers	Not explicitly reported

#### Nostril Height

Similarly, all four studies evaluated the effect of nasal conformers on nostril height ( Table 4 ) as a key parameter. Three studies showed significant improvements in nostril height among patients using nasal conformers. In contrast, Chang et al. found that the cleft side nostril height remained shorter compared with the non-cleft side, underscoring variability in outcomes among studies. 18

#### Symmetry Ratios

Two studies evaluated nostril width and height ratios as measures of symmetry ( Table 4 ). Chang et al. 18 reported ratios closer to 1, indicating better symmetry, while Funayama et al. 19 observed significant differences in nostril height ratio but not in nostril width ratio, suggesting variable effectiveness of nasal conformers in achieving overall symmetry.

## Discussion

The four selected cohorts yielded significant empirical data from cleft centers across the globe, which can guide the implementation of multidisciplinary cleft treatment protocols. Our findings suggest that nasal conformers may play an important role in improving nasal symmetry postprimary cheiloplasty, particularly in terms of nostril width and height. These results highlight the potential utility of nasal conformers across different parameters, contributing to enhanced cleft care quality globally.

The meta-analysis revealed significant improvements in nostril width and nostril height favoring the cleft side; however, these findings should be interpreted cautiously. While these improvements suggest potential benefits for nasal symmetry, their clinical relevance remains uncertain and warrants further investigation in the context of patient satisfaction and quality of life. Improved nasal symmetry may reduce the need for secondary surgical revisions, enhance aesthetic outcomes, and support psychological well-being, particularly during critical developmental stages of childhood. However, the lack of long-term follow-up data and patient-reported outcomes limits the ability to confirm these benefits.

Maintaining nasal symmetry over time remains challenging, as relapse of nasal asymmetry is often observed during growth. Although nasal conformers appear to support corrected nasal structures postoperatively, their long-term impact on symmetry is unclear due to the lack of standardized follow-up protocols in the included studies. Relapse may serve as a physiological mechanism to balance overexpansion rather than achieving permanent symmetry. 11 18 This challenge is particularly pronounced in Asian populations, where small, underdeveloped alar cartilage and thick skin contribute to a higher relapse rate after corrective surgery. 22

The challenges associated with nasal conformers, such as intricate production processes, inconsistent retention, risk of pressure sores, and high costs, 23 24 25 underscore the need for innovation and adaptation. These challenges may impact adoption differently in high-resource and low-resource settings. High-resource settings could overcome production barriers through advancements in 3D printing and customization, while low-resource settings might benefit from simplified, cost-effective alternatives. Addressing these disparities is crucial for ensuring global accessibility and equity in cleft care.

The evidence base for this meta-analysis is limited by the small number of studies included, none of which were RCTs. While ethical and logistical constraints often preclude RCTs in pediatric populations, the variability in study design and outcome measures limits the robustness of the findings. Furthermore, the absence of long-term evaluations and standardized anthropometric assessments for nasal symmetry complicates the interpretation of results and comparison across studies. These limitations highlight the need for cautious interpretation and emphasize the exploratory nature of this analysis.

Future research should aim to address the gaps identified in this review by focusing on standardized nasal anthropometric measures and patient-reported outcomes for assessing both symmetry and quality of life. Prospective studies are needed to evaluate the durability of nasal symmetry improvements and their impact over time. Exploring diverse study designs, such as pragmatic clinical trials or well-controlled cohort studies, could strengthen the evidence base. Furthermore, technological innovations such as advancements in 3D imaging and printing hold promise for creating customized nasal conformers tailored to individual anatomical differences.

Compliance remains a significant issue, particularly in younger patients, as adhesive tapes used to secure conformers can lead to parental frustration and reduced effectiveness. 26 Despite these challenges, compliant use of nasal conformers in infants undergoing presurgical molding and complete cleft lip repair has been associated with improved outcomes, higher satisfaction with nostril shape, and reduced scar contracture. 27 Nasal conformers help maintain or slightly increase cleft-side nostril height by supporting the nasal alar and compensating for scarring. 23 28 Although the cleft-side nostrils are generally wider due to premaxilla projection, this is often less noticeable from the frontal view and has minimal psychological impact. Patients with a unilateral cleft lip are advised to use nasal conformers for at least 6 months postsurgery to maintain corrected nasal structures and improve aesthetic outcomes. 29 Overcorrection of up to 20% is recommended to preserve nostril height at age 5, ensuring sustained benefits. 9 11

This study suggests that nasal conformers may improve nasal symmetry in patients with UCLP after primary cheiloplasty. These findings provide a foundation for future research to define nasal symmetry using objective anthropometric measurements and evaluate long-term outcomes. Prospective randomized clinical trials and technological advancements in 3D imaging and printing hold promise for developing more effective and accessible nasal conformers.
