# Supplementary material for: The Effectiveness of Nasal Conformers in Patients with Unilateral Cleft Lip and Palate Following Primary Cheiloplasty: A Systematic Review and Meta-Analysis
Source: Arch Plast Surg. 2025 Jul 23;52(4):225–33. doi: 10.1055/a-2572-6342 (PMC12286684; doi:10.1055/a-2572-6342)
Supplement: Supplementary file 1 — Supplementary Material [file 10-1055-a-2572-6342_s24jul0118oa.pdf]

Supplementary Table S1 Search strategy

Number	Search term	Search results			
		PubMed	EMBASE	CENTRAL	EBSCO Open Dissertation
1	"Cleft lip"	21,421	27,268	846	234
2	"Cleft lip"[MeSH]	17,195	19,268	370	1
3	"Hare lip"	167	154	2	9
4	"Mouth cleft"	15	12	0	7
5	"Oral cleft"	376	465	20	19
6	"Orofacial cleft"	716	872	12	810
7	Stent	139,811	244,167	15,396	385
8	"Stents"[MeSH]	90,523	219,497	5,641	0
9	Conformer	727,908	6,418	25	12,147
10	Retainer	3,239	2,093	306	509
11	Creator	1,946	1,084	44	13,337
12	OR/1–6	21,693	27,603	855	2
13	OR/7–11	872,747	258,061	16,911	234
14	AND/12–13	255	218	16	1

Abbreviation: CENTRAL, Cochrane Central Register of Clinical Trials.

Supplementary Table S2 List of excluded studies after full-text review

Number	Reference
1. No outcome of interest (six studies)	
1.	Yeow VK, Chen PK, Chen YR, Noordhoff SM. The use of nasal splints in the primary management of unilateral cleft nasal deformity. Plast Reconstr Surg. 1999;103(5):1347–1354.
2.	Bezuhly M. Rapid intraoperative fabrication of an inexpensive, reliable nasal stent for use after primary cleft nasal repair. Cleft Palate Craniofac J. 2014;51(5):e110–e112.
3.	Bhutiani N, Tripathi T, Verma M, Bhandari PS, Rai P. Assessment of treatment outcome of presurgical nasoalveolar molding in patients with cleft lip and palate and its postsurgical stability. Cleft Palate Craniofac J. 2020;57 (6):700–706.
4.	Hennocq Q, Person H, Hachani M, Bertin H, Corre P, Gorbonosov V, et al. Quality of life and nasal splints after primary cleft lip and nose repair: Prospective assessment of information and tolerance. J Craniomaxillofac Surg. 2018;46(10):1783–1789.
5.	Rossell-Perry P, Romero-Narvaez C, Gavino-Gutierrez A, Figallo-Hudtwalcker O. Postoperative nasal conformers in cleft rhinoplasty: Are they efficacious? J Craniofac Surg. 2023;34(5):1416–1419.
6.	Tang PM, Chao NS, Leung MW, Kelvin KW. Changes in nasal configuration following primary rhinoplasty: Direct anthropometric measurement in patients with complete unilateral cleft lip and palate. Cleft Palate Craniofac J. 2016;53(5):557–561.
2. Not RCT or cohort study (four studies)	
1.	Bajaj A, Shetty V, Pahwa I, Bajaj M. The use of a simplified nasal stent in infants with complete unilateral cleft lip and palate. J Oral Maxillofac Surg. 2012;70(7):e415–8.
2.	Chauhan JS, Sharma S. A simple and economical nasal conformer for clefts! J Maxillofac Oral Surg. 2021;20 (1):157–159.
3.	Luo D, Li T, Wang H, Chen Y. Three-dimensional printing of personalized nasal stents for patients with cleft lip. Cleft Palate Craniofac J. 2019;56(4):521–524.
4.	Yuzuriha S, Matsuo K, Kondoh S. A newly designed nasal retainer to sustain the corrected shape of the nostril rim and anterior nasal recess for cleft lip patients. Plast Reconstr Surg. 2001;108(2):452–455.

Abbreviation: RCT, randomized controlled trial.

 © 2025. The Author(s).
